# Interactive Toolkit for Classifying Digital Health Interventions, Services, and Applications Based on the WHO Framework

**DOI:** 10.1007/s10916-025-02172-5

**Published:** 2025-04-07

**Authors:** Anuradha Liyanage, Daniela Wurhofer, Mahdi Sareban, Gunnar Treff, Josef Niebauer, Rada Hussein

**Affiliations:** 1 Ludwig Boltzmann Institute for Digital Health and Prevention, Salzburg, Austria; 2 University Institute of Sports Medicine, Prevention and Rehabilitation, Paracelsus Medical University, Salzburg, Austria; 3 Institute of Molecular Sports Medicine and Rehabilitation, Paracelsus Medical University, Salzburg, Austria

**Keywords:** Digital health interventions, WHO classification, Toolkit development, Health system challenges

## Abstract

The rapidly advancing digital health requires a standardized approach to classifying Digital Health Interventions (DHIs) for better planning, monitoring, and resource distribution. The World Health Organisation (WHO) developed a Classification for Digital Health Interventions, Services, and Applications in Health (CDISAH) in response to this need. The purpose of this study was to develop an interactive toolkit based on WHO's CDISAH to enhance categorization, making it more interactive, user-friendly, and effective in classifying DHI services and applications, and demonstrate its practical implementation in the field of cardiac rehabilitation. We used a descriptive approach with a seven-step iterative process to create the toolkit. The process began with a review of best practices for converting framework into toolkit, followed by drafting an initial toolkit structure, which was refined through team discussions. The content was based on WHO CDISAH. Expert feedback was incorporated, and quality assurance was conducted through internal and external reviews. The toolkit’s functionality and usability were evaluated through a use case including DHIs, services, and applications for cardiac rehabilitation. The toolkit for WHO CDISAH has a structured interface with clear definitions, practical examples, and intuitive navigation across three main axes: health system challenges, digital health interventions, and digital health applications and services. Pilot testing improved its usability and functionality for accurate classification, highlighting areas for refinement and identifying challenges and solutions for practical implementation. The developed toolkit provides a standardised, portable platform for classifying the multimodal DHIs that align with the framework presented by WHO.

## Introduction

Digital health has been demonstrating benefits in offering better healthcare service [1], patient empowerment [2], and expanding access to underserved populations through remote diagnostics and monitoring [3]. It enhances the quality of care through timely and coordinated access to patient medical histories and optimizes resource utilization through remote patient monitoring to lessen the workload of medical staff [4, 5]. Effective digital healthcare implementation can optimize resource utilization while strengthening the delivery of high-quality medical treatment [6, 7]. Since the COVID-19 pandemic significantly interrupted in-person services, the delivery of health care via digital means has gained popularity [8]. Innovation in digital health has advanced at a constant and rapid rate [6–9], resulting in the creation, application, evaluation, and improvement of novel therapies for a range of situations and challenges. These services help individuals, health workers, and system users manage problems and enhance access to high-quality information and medical actions [7]. Consequently, classifying digital health interventions is a necessary step for conducting and evaluating inventories of existing assets and for identifying gaps, preventing duplication, and effective planning and resource allocation [10].

Classifying Digital Health Interventions (DHIs) provides a standardized vocabulary and framework that improves communication among healthcare practitioners, policymakers, technologists, researchers, and funders, ensuring uniform understanding and cooperation. It also facilitates the essential systematic evaluation and data collection for scaling successful interventions and developing legal and regulatory frameworks and supporting comprehensive, comparable academic studies. Additionally, it allows for monitoring trends and advancements in digital health and adjusting action plans as needed [10].

**Fig. 1:**
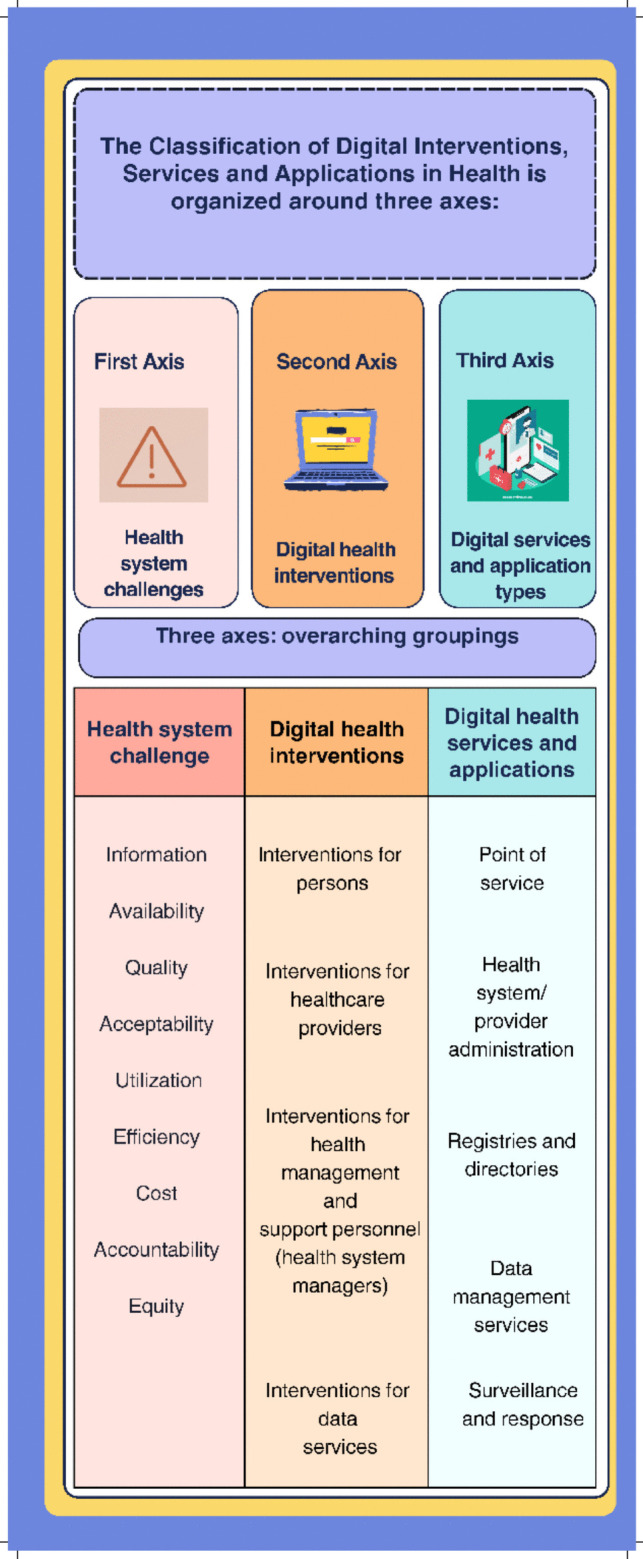
WHO’s three main axes of the Classification of Digital Interventions, Services and Applications in Health [11]

Recognizing the need for a structured approach to categorizing these diverse digital health interventions, the World Health Organization (WHO) developed the first edition of the Classification of Digital Health Interventions (CDHI) in 2018 [10] and a new edition in 2023 [11]. A collection of classifications known as the revised Classification of Digital Interventions, Services and Applications in Health (CDISAH) connects the ways in which DHIs integrated into digital applications and services are used to meet the requirements and problems of the individual and the health system. This classification system, known as a taxonomy, is arranged along three axes: namely, health system challenges, digital health interventions, and digital health applications and services (see Fig. 1).

The WHO and its partners have developed several resources [12–16], including this CDISAH classification [11], to aid in the planning and execution of a digital health enterprise. The classification is comprehensive and includes detailed explanations and examples. It also introduced four use cases. Use case one involves implementing DHI(s) to address a health system challenge. Use case two focuses on articulating the DHI(s) delivered by services or applications. Use case three examines the landscape analysis of digital health technology investment. Use case four involves searching the WHO Digital Health Atlas (DHA) for registered projects. To fully leverage the value of this extensive classification, a more user-friendly and easily accessible tool is needed.

This work aimed to create an interactive and user-friendly toolkit to classify digital interventions, services, and applications in health according to the WHO framework. We target the use case two, which articulates the DHI(s) delivered by services or applications to provide a concrete example based on practical implementation in the cardiac rehabilitation domain.

## Methodology

This work describes the iterative process with seven iterative steps of developing the toolkit. We performed an in-depth examination to explore the steps involved in the toolkit's creation [17] using the standards for reporting of health informatics guidelines [18].

### Step 1-Preliminary search on converting guidelines into a toolkit

**Fig. 2:**
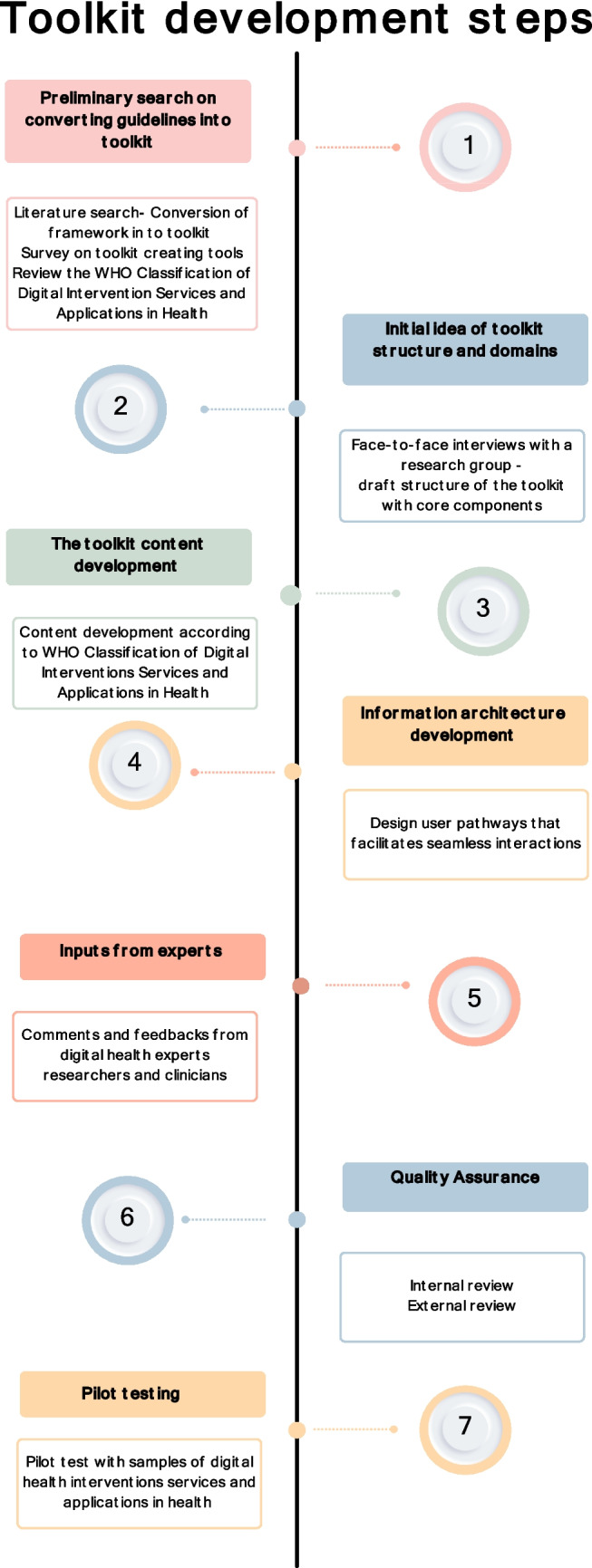
Steps of Classification of Digital Health Interventions applications and services toolkit development

We conducted a preliminary search on how the framework and guidelines could be converted into a toolkit. We identified best practices, reviewed existing toolkits [19, 20] and analyzed their structures. We also explored several resources for toolkit development guides [21, 22]. Accordingly, a pragmatic, iterative process for developing a toolkit was identified and adopted (see Fig. 2).

Moreover, we conducted a thorough analysis of the underlying concepts and criteria used by the WHO in its CDISAH [10, 11] to improve understanding of their application. A number of toolkit creation tools were assessed, including ‘Fillout’ forms, Excel, specialized software platforms like Google Forms, and others.

### Step 2: Initial idea of toolkit structure and domains

Drawing from the literature review on the WHO portal for digital health guidelines with special emphasize on WHO CDISAH, we initially formed the structure consisting of several core components. After conducting face-to-face interviews with a research group consisting of two digital health informatics experts, three clinicians, and one public health personnel, the core components and overall structure of the toolkit were refined and drafted.

The interviews focused on three main areas: proposing a toolkit structure, the classification domains, and suggestions for the user interface and functionality. The insights gathered from these discussions were instrumental in refining both the core components and the overall structure of the toolkit.

### Step 3: The toolkit content development

We created detailed and accurate content based on the WHO CDISAH and other WHO guidelines [10, 11] using the ‘Fillout’ online platform (https://www.fillout.com/).

The process involved a thorough review of these documents to extract key concepts, definitions, classification criteria, explanations, and examples that would be most useful in a practical toolkit.

### Step 4: Information architecture development

The aim of this phase was to organize and structure the content to maximize usability and efficiency. This involved creating a logical and intuitive framework for the toolkit, ensuring that users can easily access and navigate through the various components. Key elements of this phase included defining the hierarchy of information, categorizing the classification criteria, and determining the optimal layout for definitions and examples. Additionally, attention was given to designing user pathways that facilitate seamless interaction with the toolkit, including clear instructions, user-friendly menus, and accessible help resources [23, 24].

### Step 5- Inputs from experts

The toolkit was refined through valuable insights and feedback from a diverse panel of experts who were not part of the author team, including three digital health experts, three clinicians, and five researchers. They evaluated the toolkit’s components, functionality, ease of use, and applicability. This process involved collating the insights to pinpoint specific areas of improvement and validate the strengths of the existing design.

### Step 6- Quality Assurance

The toolkit underwent a critical phase of quality assurance to ensure it met accuracy in terms of alignment with the WHO framework. This involved:

Internal Review: The research team conducted an internal review to ensure the toolkit aligned with the project objectives and included all essential components of the WHO CDISAH.

External Review: Three external experts in digital health were consulted to provide independent feedback on the toolkit. Their feedback helped to improve the presentation, including navigation, usability issues (such as how to access previous sections), the user guide, font size, and colour choices. Their insights were instrumental in enhancing the overall user experience and usability of the toolkit.

### Step 7- Pilot testing

This phase aimed to assess the practicality and effectiveness of the developed toolkit. We expanded the use case 2 in the WHO CDISAH [11] with the practice implementation into cardiac rehabilitation field. For this purpose, we collated five applications from the field.

1. Aktivplan; (https://dhp.lbg.ac.at/app-development-aktivplan/?lang=en-) The ‘Aktivplan’ application is a digital tool designed to assist healthcare professionals and patients in planning, monitoring, and optimizing heart-healthy physical activity plans. It functions as both a web and mobile application, facilitating collaboration between patients and health professionals. Patients work with experts to set up personalized activity plans, choosing exercises they enjoy and setting meaningful goals. The application allows patients to log activities, access exercise resources, and review their progress via a calendar interface. Health professionals can monitor patient activity through the application, export logs for medical records, and use the data to guide ongoing patient care.
2. MORE (Modular Open Research Platform for Digital Health; https://dhp.lbg.ac.at/more/?lang=en)—The MORE Platform is a digital infrastructure project designed to support sustainable health research by streamlining the creation and execution of studies using digital technologies.
3. HERO (Heart Rehabilitation Information Tool; https://dhp.lbg.ac.at/hero-das-herz-reha-informationstool/?lang=en)—The HERO application aims to bridge the information gap by providing clear guidance on how to get referrals for cardiac rehabilitation and offering other essential information related to follow-up care.
4. Active Waiting—This digital application provides a quick-and-easy tool that can be used to fill waiting and/or break times with spontaneous bouts of physical activity.
5. Shared Achievements – The Shared Achievements digital application focuses on shared contributions of group members into pooled activity outcome achievements and their communication (e.g. visualization) to other team members.

A qualitative data collection method was employed to gather comprehensive insights during the pilot testing phase. A total of five in-depth interviews were conducted, one for each selected digital health application. Responsible contact persons of each selected intervention, application or service were interviewed for the exploration of diverse perspectives on the toolkit’s functionality and applicability. The interviews explored several core areas, including:

(i) The ease of use and clarity of the toolkit in accurately classifying digital interventions
(ii) Practical challenges were encountered during the use of the toolkit, such as issues with the user interface
(iii) Suggestions for improvement were gathered, especially concerning the instructional guide and support provided for users.
(iv) The relevance of the WHO classification framework to the specific interventions being used and how well the toolkit aligned with their needs

## Results

**Fig. 3:**
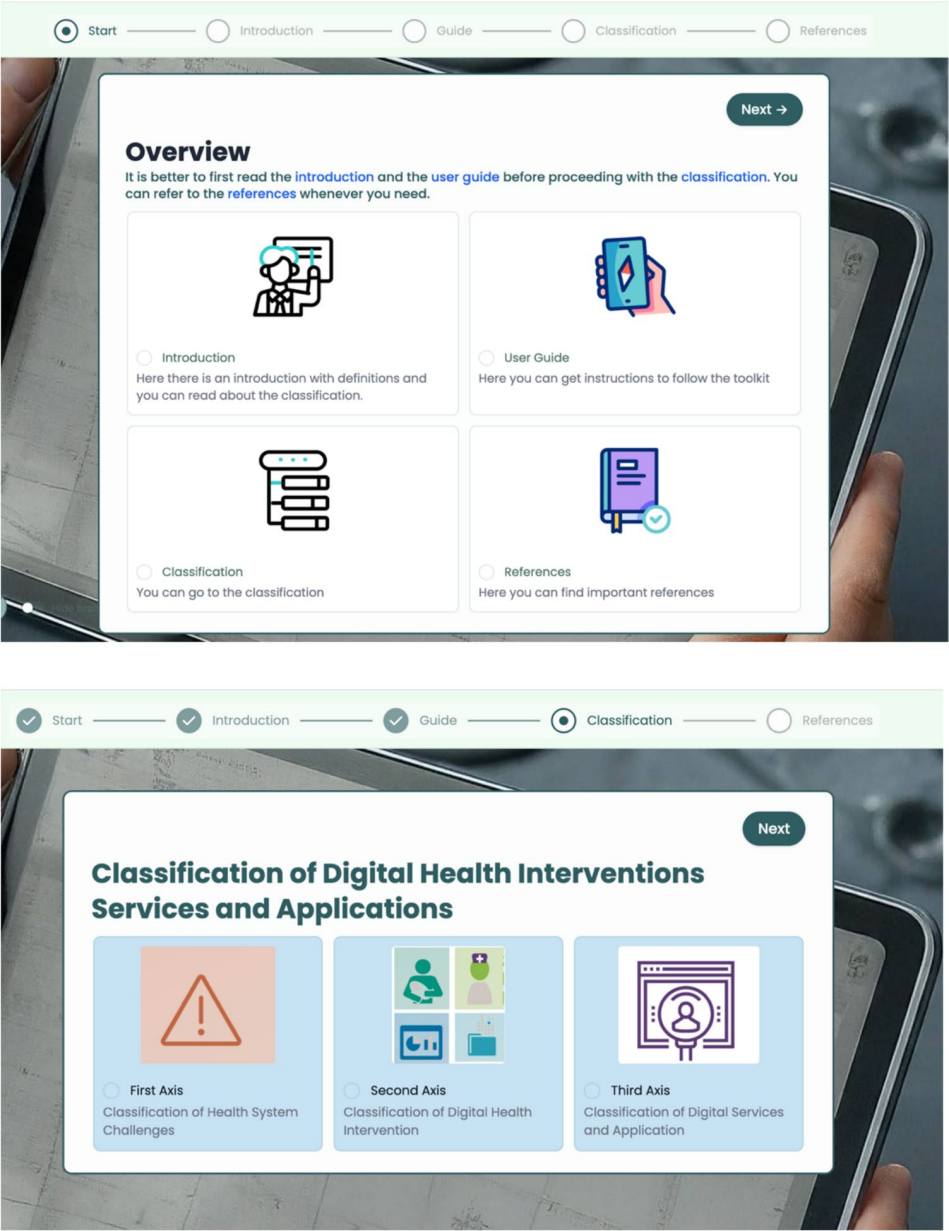
Domains of the toolkit for Classification of Digital Health Interventions Services and Applications

The toolkit's content was meticulously developed to ensure clarity and accuracy in classifying DHIs services and applications (see Fig. 3). The toolkit was organized into four main sections: introduction, guide to use, classification, and references. The introduction section provides important definitions related to the classification of DHIs and applications and services, an introduction to the toolkit for CDISAH, and a guide to using the toolkit. The toolkit is available for use and can be accessed via (https://forms.fillout.com/t/1hjbK9pz6gus).

Classification criteria were defined clearly and precisely, adhering to WHO CDISAH three axes. Detailed explanations and practical examples were included to illustrate the application of each criterion, thereby enhancing user understanding and consistency in classification. Visual elements were added throughout all axes for better comprehension and usability [23, 24]. A summary map of the classifications was generated on the summary page and could be emailed to the user's specified email address.

**Fig. 4:**
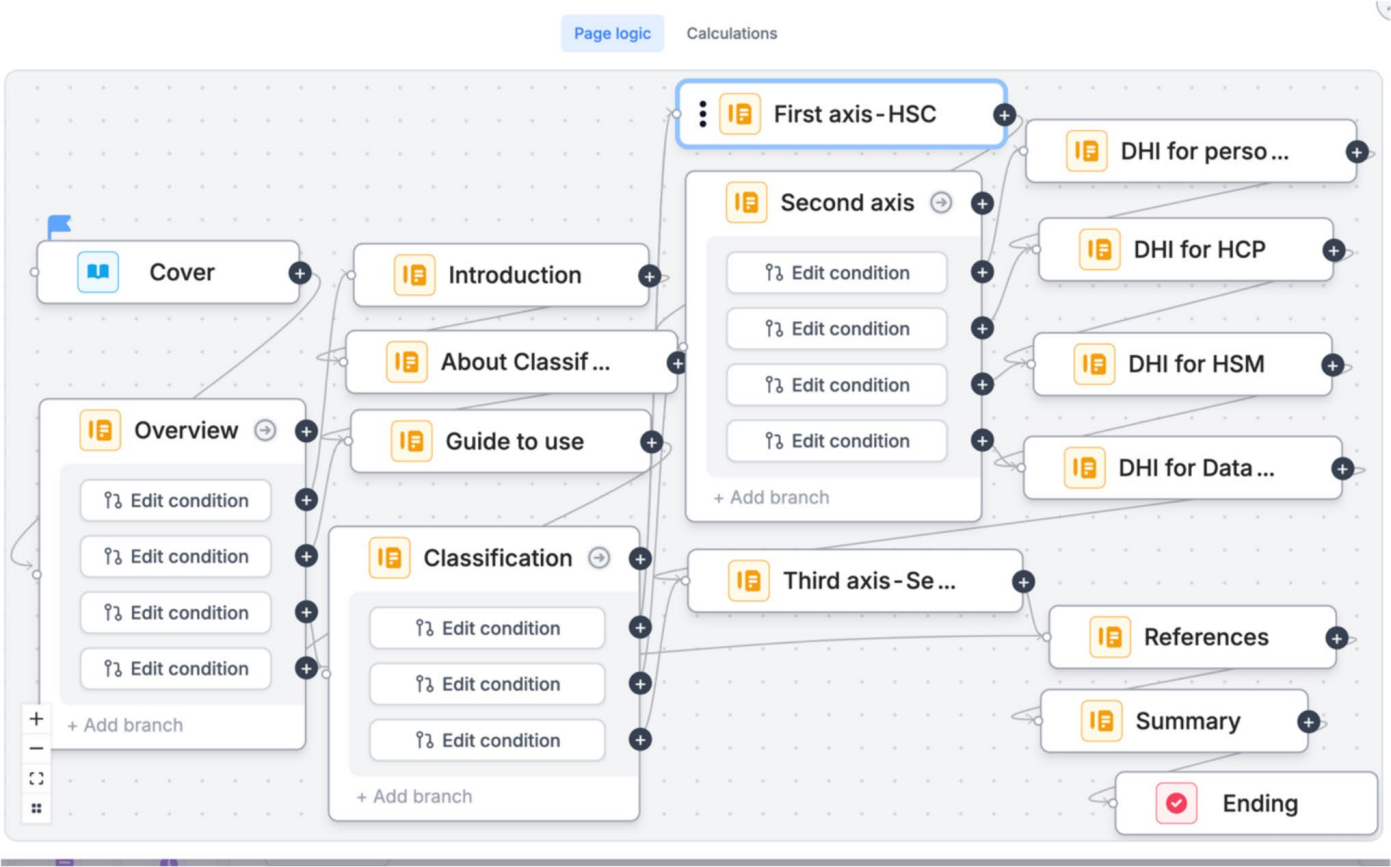
Navigation architecture among interfaces of the toolkit

The toolkit's logical and intuitive framework organizes content hierarchically, ensuring ease of navigation and accessibility for users (see Fig. 4).

Experts found the toolkit highly applicable to various healthcare settings, noting the benefit of generating summary reports for policymakers, though some recommended adding more customizable options for specific regional use cases. While clinicians and researchers commended the toolkit’s intuitive design, it was suggested that more comprehensive onboarding materials or tutorials be included for users with limited digital health experience. Based on these insights, refinements were made to improve navigation, add explanatory content, and enhance the user guide for broader accessibility.

### Results of the pilot testing and reporting of the cardiac rehabilitation use case

**Fig. 5:**
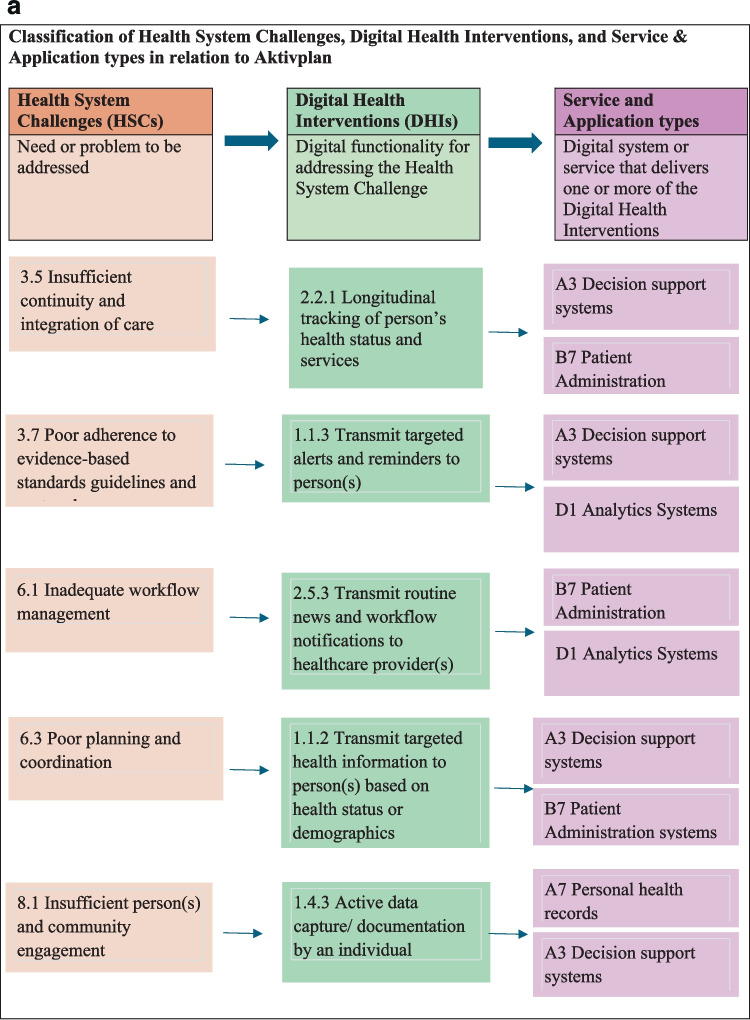

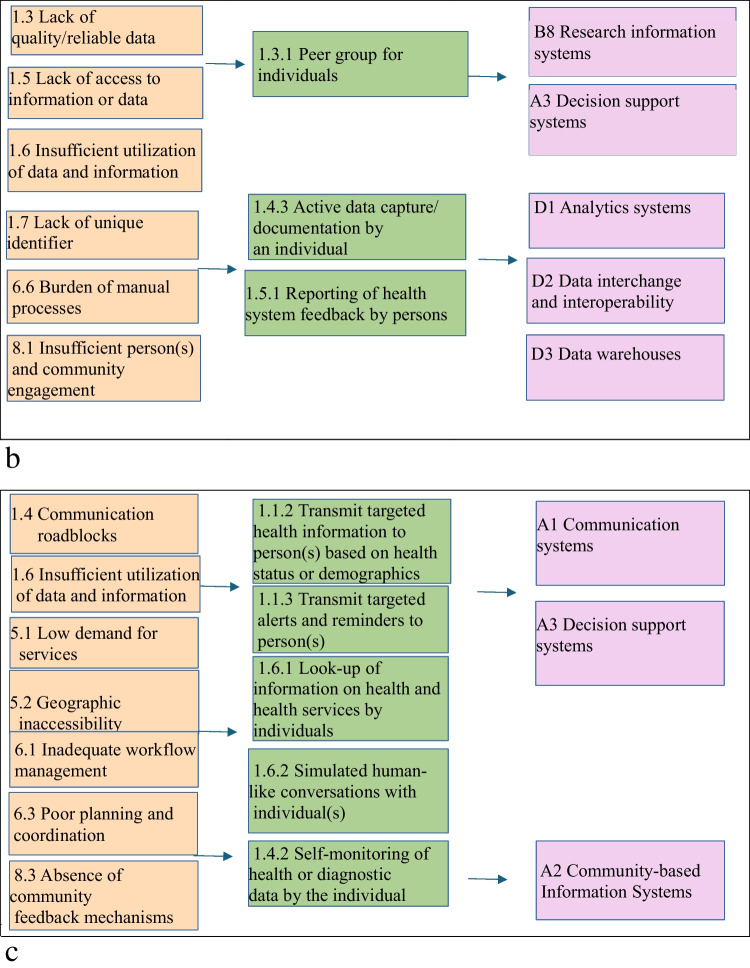

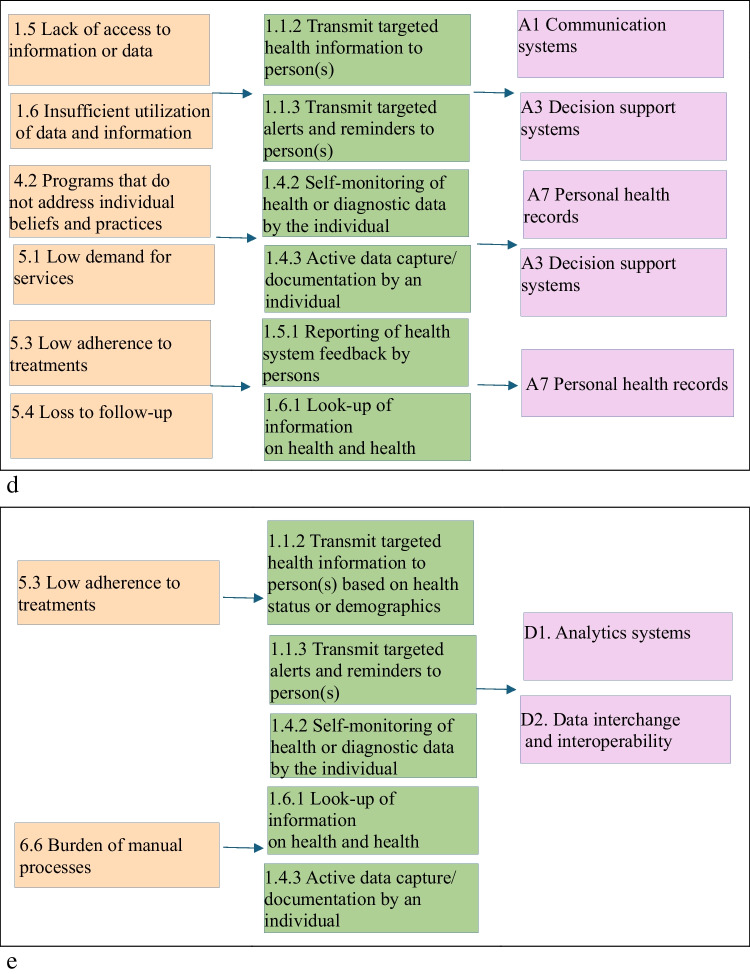
**a** Linkages across health System challenges, digital health interventions and Aktivplan application **b** Linkages across health system challenges, digital health interventions and MORE **c** Linkages across health system challenges, digital health interventions and shared achievements. **d** Linkages across health system challenges, digital health interventions and HERO **e** Linkages across health system challenges, digital health interventions and Active waiting

A summary of the classification of five examples in the cardiac rehabilitation according to CDIASH is shown in Fig. 5.

The participants of the pilot testing appreciated the clear purpose of the toolkit, which effectively guided them in classifying DHIs.

When comparing the WHO document and the tool, users generally appreciate the comprehensive and detailed nature of the WHO framework but find the step-by-step structure and ease of use provided by the tool to be more efficient and accessible for practical application. One participant noted,

“The WHO Digital Health Intervention (DHI) framework is comprehensive and highly informative, providing in-depth classifications that cover a wide range of digital health interventions. However, its format resembles a book, requiring users to frequently go up and down the pages or refer back to previous sections, which can be time-consuming and cumbersome. In contrast, my tool offers a more user-friendly experience: it allows users to proceed step-by-step, skip unnecessary steps, and directly read only the relevant examples and explanations.”

Overall, participants reported that the toolkit was intuitive and easy to navigate. The clarity of the classification criteria was frequently praised. One participant noted,

"*The criteria were clearly defined, which made it simple to classify our digital health interventions."*

They found the inclusion of practical examples particularly beneficial for enhancing understanding and application of the WHO classifications. One user stated,

*"The inclusion of examples was particularly beneficial in helping me grasp the framework."*

The picture choices were eye-catching and efficiently conveyed concepts. The capability to receive a summary at the end, along with the option to email the summary and create a PDF document, was positively reviewed.

However, participants suggested several improvements to enhance the toolkit. For instance, they recommended that the starting page clearly labeled the resource as a "toolkit" and articulate its purpose on the first page to improve user understanding. One user commented,

*"I wasn’t sure what the initial page was meant for—it should be clearer."*

This indicates a need for better communication regarding the toolkit's intent.

Participants also suggested ensuring consistent terminology in the top menu to enhance coherence. Another suggestion was to make the "Next" button visible without scrolling and allow direct navigation to categories. Additionally, they recommended adding guidance to indicate that a selection must be made before clicking "Next," along with an improved error message for clarity. One user noted,

*"It was frustrating to have to scroll down to find the 'Next' button,"* indicating a need for improved navigation usability.

Improving navigation options, allowing users to easily return to previous pages, and ensuring the top menu functions properly were also identified as key areas for enhancement. A participant commented,

*"On the overview page, I couldn't tell what I should select first or if it was parallel pathways without any connection.*"

Further recommendations included reducing excessive scrolling by utilizing wider text boxes and adjusting colors for better accessibility, particularly for users with color blindness. While the tool is convenient for step-by-step classification, one user commented:

“*It may lack the flexibility of the WHO document's narrative approach, which allows for more extensive interpretation and explanation of digital health interventions. The tool might struggle with highly complex or multifaceted interventions that don't fit neatly into predefined categories. In such cases, the more detailed WHO framework may offer a better understanding of the nuances*”.

During the classification of digital health interventions, several practical challenges emerged. Certain applications exhibited multi-purpose capabilities, making it difficult to assign them a single classification. For example, the MORE Platform functions as a research information system while also supporting data management services. Similarly, Aktivplan serves both as a planning tool and a behavioral health management system. In that circumstance the primary function was prioritized while acknowledging secondary functionalities. This ensured that applications were not misclassified based on a singular feature.

The application's potential to support patient adherence to rehabilitation or prescribed activity plans blurred the lines between personal use and clinical health monitoring, making its classification dependent on use-case context. To address these challenges, Aktivplan was categorized under Personal Health Tracking (1.4), and also under targeted communication to person (1.1) if used in clinical pathways. This approach ensures its classification reflects both general self-monitoring and potential clinical utility.

Applications such as Active Waiting and Shared Achievements promote physical activity and motivation but do not include direct clinical interventions. This made it challenging to differentiate them from apps used in rehabilitation or medical treatment. Consequently, we classified them under Personal Health Tracking (1.4), as they promote general activity rather than structured medical exercise.

A key challenge in classifying the HERO application was distinguishing between targeted health communication and patient self-management, as it provides both structured education and interactive guidance. Another challenge was determining whether it functions as a passive information tool or an active decision-support system, as it helps users navigate rehabilitation but does not fully integrate with electronic medical records. To address these issues, the application was classified primarily under Targeted Communication to Persons (1.1.2) and On-Demand Communication (1.6.1) for its informational role, while also recognizing its Personal Health Tracking (1.4.2).

Based on pilot testing feedback, several key enhancements were made to the toolkit's usability and user experience. Clear guidance was added for required selections, along with improved error messages. The overview page was reorganized for logical navigation, while layout adjustments reduced excessive scrolling. Accessibility was enhanced with better color contrast for users with vision deficiencies. The top menu was refined for better functionality, allowing users to return to previous pages easily. Finally, the summary feature was improved for clearer formatting and easier sharing via email and PDF export.

## Discussion

This conversion of the WHO framework into a manageable toolkit improves usability and accessibility, enabling a range of stakeholders to effectively use the WHO classification criteria in real-world scenarios. Thus, the development of the interactive toolkit is crucial for streamlining workflows and facilitating collaboration across various domains, ultimately driving innovation and improving outcomes. This will also ensure consistent understanding and collaboration among diverse stakeholders, such as healthcare professionals, policymakers, technologists, researchers, and funders, by providing a standardized vocabulary and structure.

The CDISAH toolkit aligns with the WHO framework to help identify gaps in existing digital health initiatives and avoid duplication, optimizing resource allocation. It ensures that digital health efforts target unmet needs, supports evidence-based decision-making, and assists in developing strong regulatory frameworks. Additionally, the toolkit aids in creating national and subnational inventories, guiding investment decisions, and advancing digital health education and training.

The use case on cardiac rehabilitation revealed several critical insights into the toolkit’s usability and functionality. Participants frequently expressed confusion regarding the toolkit’s purpose and navigation, prompting revisions to clarify its identity and streamline user pathways. Accessibility improvements, particularly regarding color contrast, underscore the toolkit's commitment to inclusivity. Positive feedback on the summary feature indicated that users appreciate having a clear, shareable format for their results, which may enhance engagement and satisfaction. The use case on cardiac rehabilitation offered valuable insights into the practical implications of digital health interventions, which are designed to be delivered through applications related to cardiac rehabilitation (see Table 1) by classifying DHIs.

**Table 1:** Digital health interventions delivered by digital health applications in relation to cardiac rehabilitation

Digital health interventions	Digital health application in relation to cardiac rehabilitation
1.1.2 Transmit targeted health information to person(s) based on health status or demographics	Aktivplan, Shared Achievements, HERO, Active Waiting
1.1.3 Transmit targeted alerts and reminders to person(s)	Aktivplan, Shared Achievements, HERO, Active Waiting
1.3.1 Peer Group for individuals	MORE
1.4.2 Self-monitoring of health or diagnostic data by the individual	Shared Achievements, HERO, Active Waiting
1.4.3 Active data capture/ documentation by an individual	Aktivplan, MORE, HERO, Active Waiting
1.5.1 Reporting of health system feedback by persons	MORE, HERO
1.6.1 Look-up of information on health and health services by individuals	Shared Achievements, HERO, Active Waiting
2.2.1 Longitudinal tracking of person’s health status and services	Aktivplan
2.5.3 Transmit routine news and workflow notifications to healthcare provider(s)	Aktivplan

Looking ahead, further improvements could focus on integrating more interactive features and conducting ongoing user testing to refine the toolkit further. The pilot testing process has been invaluable in ensuring that the toolkit meets user needs, underscoring the importance of continuous feedback in developing effective digital health interventions. We plan to develop a more tailored toolkit that aligns closely with WHO guidelines, enhancing its applicability and effectiveness in practice.

## Limitations

The pilot testing phase, while comprehensive, was limited to a selected sample of five DHI services and applications. Wider testing across a more diverse range of interventions and settings would provide a more robust evaluation of the toolkit’s effectiveness and usability. To address these limitations and further enhance the toolkit’s impact, future efforts should focus on engaging in ongoing feedback loops with users and stakeholders to continually refine and update the toolkit.

## Conclusion

The user-friendly toolkit for classifying digital health interventions, services, and applications based on the WHO framework represents a significant advancement in the field of digital health. The contributions of digital health experts, clinicians, and researchers provided valuable perspectives that enhanced the toolkit’s relevance and usability.

By providing a standardized, accessible, and practical tool for classifying digital health interventions, the toolkit has the potential to enhance the planning, implementation, and evaluation of digital health initiatives globally. The use case on cardiac rehabilitation offered valuable insights into both the functionality of the toolkit and the practical implications of classifying DHIs.

## Data Availability

No datasets were generated or analysed during the current study.

## Abbreviations

CDISAH: Classification for Digital Health Interventions, Services, and Applications in Health
CDHI: Classification of Digital Health Interventions
DHI: Digital Health Intervention
WHO: World Health Organization
